# Aiming for Maximized and Reproducible Enhancements in the Obstacle Race of SERS

**DOI:** 10.1021/acsmeasuresciau.3c00037

**Published:** 2023-10-31

**Authors:** Priyanka Dey

**Affiliations:** School of Pharmacy and Biomedical Sciences, University of Portsmouth, Portsmouth PO1 2UP, U.K.

**Keywords:** SERS, hot-spots, plasmonic nanoparticles, enhancement factors, analytical EF, single molecule EF, nanoassemblies, Raman labels, resonant, nonresonant

## Abstract

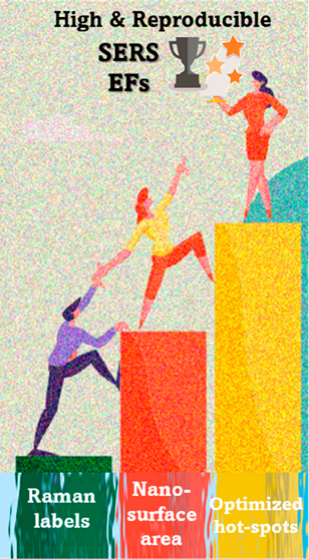

Surface enhanced Raman scattering (SERS), since its discovery in the mid-1970s, has taken on many roles in the world of analytical measurement science. From identifying known and unknown chemicals in mixtures such as pharmaceutical and environmental samples to enabling qualitative and quantitative analysis of biomolecules and biomedical disease markers (or biomarkers), furthermore expanding to tracking nanostructures in vivo for medical diagnosis and therapy. This is because SERS combines the inherent power of Raman scattering capable of molecular species identification, topped with tremendous amplification in the Raman signal intensity when the molecule of interest is positioned near plasmonic nanostructures. The higher the SERS signal amplification, the lower the limit of detection (LOD) that could be achieved for the above applications. Therefore, improving SERS sensing efficiencies is vital. The signal reproducibility and SERS enhancement factor (EF) heavily rely on plasmonic nanostructure design, which has led to tremendous work in the field. But SERS signal and EF reproducibility remain key limitations for its wider market usability. This Review will scrutinize factors, some recognized and some often overlooked, that dictate the SERS signal and are of utmost importance to enable reproducible SERS EFs. Most of the factors pertain to colloidal labeled SERS. Some critically reviewed factors include the nanostructure’s surface area as a limiting factor, SERS hot-spots including optimizing the SERS EF within the hot-spot volume and positioning labels, properties of label molecules governing molecule orientation in hot-spots, and resonance effects. A better understanding of these factors will enable improved optimization and control of the experimental SERS, enabling extremely sensitive LODs without overestimating the SERS EFs. These are crucial steps toward identification and reproducible quantification in SERS sensing.

## 1 Introduction

**Figure 1:**
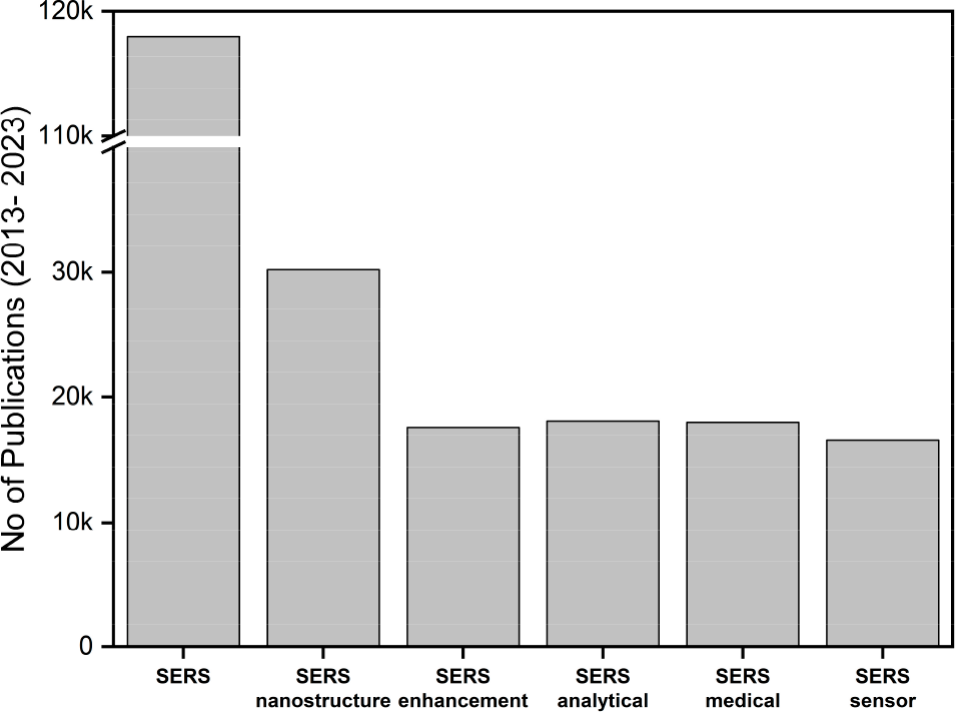
Publication metrics in SERS subdomains. (Source: Google Scholar.)

As we celebrate 50 years of discovery of the surface enhanced Raman scattering (SERS) phenomenon, it becomes eminent to discuss our current limitations and critically evaluate ways to overcome them. The popularity of SERS stems from the combined molecular fingerprinting capability of the sample-of-interest (known, unknown, toxins, biomolecule, etc.) and the tremendous enhancement of the inherent Raman signal of the sample-of-interest when brought within nanoscale proximity of plasmonic nanostructures. A higher SERS signal enhancement translates into a lower concentration i.e., improved Limit of Detection (LOD) for sensing applications and makes for better chemical and biomedical sensors.^1−3^ This allows for both a qualitative identification and quantitative estimation, vital in sensing. Although SERS enhancements enabling single molecule detection have been reported multiple times in the literature, a commercial SERS sensor has still not been accepted in the sensor market. To understand this better, the published works were categorically analyzed in the last 10 years, and a total of 118,000 publications were found with the keyword “SERS”. Also, a similar research focus can be observed in “SERS analytical” and “SERS medical” applications. Surprisingly, the number of publications for SERS enhancement and SERS sensor is almost half the number in SERS nanostructure, constituting about 25% of the total SERS publications (see Figure 1). Such a scenario is a result of (i) insufficient standardization in the SERS methods and SERS enhancement factor (EF, factor by which signal is enhanced compared to its inherent Raman signal), (ii) a stronger drive toward achieving the highest EF than the SERS or EF reproducibility, and (iii) SERS sensing being a multidisciplinary field with contributions from theoretical physicists, optical physicists, organic chemists, nanomaterial chemists, physical chemists, engineers, biologists, environmental researchers, food scientists, biomedical scientists, statisticians, and more recently machine learning experts. To drive the field toward more progress and market-acceptable sensing end-uses, a 3-fold SERS EF reproducibility is critical, including reproducible results within the same substrate or around the same nanostructure, within the same batch, and batch-to-batch. This will importantly allow SERS sensing to be considered as a quantitative rather than a qualitative or a semi-quantitative measurement,^4^ enabling SERS to compete with electrochemical sensing and optical surface plasmon resonance (SPR) sensing and outshining them due to its structural fingerprinting capabilities. This Review will critically evaluate the limiting factors affecting SERS EF’s calculation-replicability and agreement across researchers. It will also propose methodologies that could help maximize SERS EF and improve the 3-fold reproducibility, as discussed above.

## 2 Decoding SERS EF

**Figure 2:**
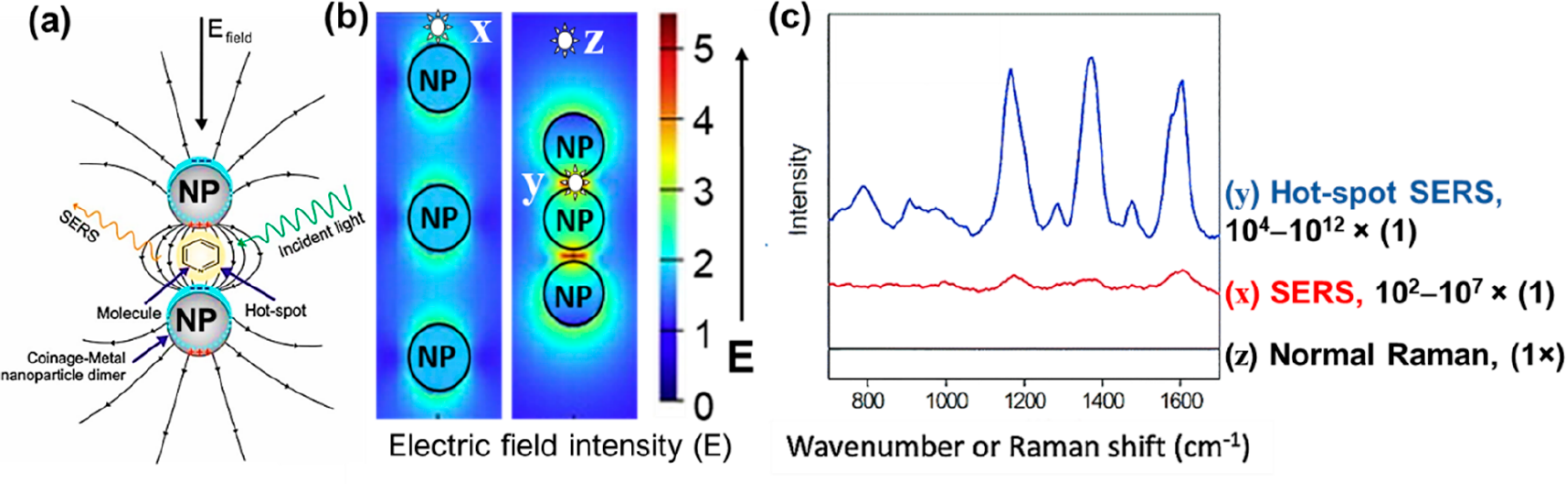
SERS phenomenon and signal amplification criteria. (a) Concept of SERS.^13^ (b) Electric field distribution around nanoparticles (NP) and at NP-NP junction hot-spots. (c) Typical SERS signal enhancements that can be observed when molecules sit at the specific virtual positions of **x**, **y**, and **z** (as shown in b). Reprinted with permission under a Creative Commons [CC-BY 4.0] from ref (13). Copyright [2021] [MDPI].

Fleischmann and co-workers first observed that the Raman signal of a molecule was significantly enhanced on an etched silver (Ag) electrode.^5^ This phenomenon was later explained with numerous experimental published works, led by van Duyne^6−8^ and termed surface enhanced Raman scattering or SERS. The primary factor for the above observation was that the plasmonic nanosurface created an electric field around it when interacting with light, and any molecule occupying such an enhanced electric field would generate an enhanced Raman signal or SERS.^6,9,10^ This is referred to as electromagnetic enhancement and is deemed as the primary contributor to SERS, with a significantly less contribution from chemical enhancement^11^ The SERS signal is dependent on where the molecule-of-interest sits in the electric field created around the plasmonic nanostructures (a cartoon depiction has been shown in Figure 2a and b) and its relative SERS signal amplification possible in Figure 2c. The three positions are marked as **z**, **x**, and **y**. Here, **z** relates to a molecule position away from a nanoparticle (NP), i.e., in a minimum electric field intensity zone resulting in a Raman (not enhanced) signal, shown as the black spectrum with significantly low signal intensities observed. In contrast, if the molecule sits at position **x** on a single NP surface, the enhanced Raman signal observed is approximately 10^2^–10^7^ times when placed at **z**. Further enhancements can be observed when the molecule is placed at NP–NP junctions which are nanozones of the intensified electric field referred to as hot-spots, as in **y**, providing a possible hot-spot SERS signal enhancement in the range of 10^4^–10^12^ times than its normal Raman.

In simplicity, when developing SERS nanostructures and aiming for the highest SERS enhancements possible, silver nanostructures are more beneficial than gold, which is more beneficial than copper and other inorganic nanostructures being investigated. The benefit of gold compared to silver for use in analytical and biomedical SERS sensing is due its chemical affinity toward functional groups allowing easy molecule (label, target analyte for detection, capture ligand etc.) attachment and functionalization, and lower toxicity than silver.^12^ This review will thus discuss both silver and gold surfaces, while focusing on gold nanostructures.

The SERS effect is most importantly quantified by the EF, which signifies the extent of amplification of the Raman signal. This EF can be substantial enough to facilitate the observation of single molecules in many instances.^14^Table 1 compiles the various forms of SERS EF calculations used by researchers in the field. However, for a considerable amount of time, the magnitude of the SERS EF has been at the center of persistent controversies in the field, with quoted values varying significantly for similar experimental conditions. The notion of SERS EFs reaching magnitudes as high as 10^14^ originated from pioneering single-molecule SERS studies, but the quoted EFs were later attributed to incorrect normalization with respect to nonresonant Raman. This misconception posed significant challenges for theorists and experimentalists attempting to justify and achieve or replicate the enormous EFs, whereas the electromagnetic calculations supported an EF of up to 10^8^–10^10^. This situation hindered progress in the field and made it challenging to develop a reliable analytical tool based on a technique with such profound discrepancies in its fundamental quantification. The hype around excessively large SERS EFs has subsided, and most overestimations of EFs can now be attributed to improper definitions or other more subtle sources of errors.^15^ Researchers are now encouraged to report more realistic EF values, such as 10^8^ or even 10^6^, which are entirely respectable figures as long as the experimental approach used to estimate them is well-detailed and justified without many assumptions. For attempting single molecule SERS detection, typical SERS substrates with larger enhancements are used but most of them may only achieve a low efficiency of SERS enhancement at the hot-spots defined as η. The values of η reported range from a low 10^–3^ to the highest of 10^–1^ when site-specific adsorption methods were employed.^16,17^ The correlation of these has been critically evaluated and is presented in section 3. By embracing accurate estimations and ensuring transparency in experimental practices, the field of SERS can continue to advance without the burden of controversies. This critical review attempts to support that journey.

**Table 1:** SERS EF Calculations and Relations^a^

analytical EF (AEF)	SERS substrate EF (SSEF)	single molecule EF (SMEF)	hot-spot efficiency for single molecule detection
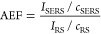	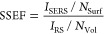	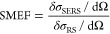	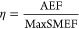

a *I* ⇒ Intensity, *c* ⇒ concentration, *N* ⇒ number of molecules, *N*_Vol_ (= *c*_RS_*V*_Sca_) ⇒ the average number of molecules in the scattering volume (*V*_Sca_) for the Raman (non-SERS) measurement, dσ*RS*/dΩ ⇒ the normal Raman cross section.

In the context of SERS detection, it is crucial to consider that the signal enhancement diminishes exponentially as the molecule of interest or target moves farther away from the plasmonic nanosurface (nanoparticle or nanostructured surface). As a general guideline, molecules positioned within 0–10 nm from the surface offer better chances of detection.^18^ This aspect holds significant importance in the discussion of SERS detection. SERS EF measurements and calculations typically employ a small Raman label (often <1 nm) and feature a high Raman cross section. These form the category of labeled SERS detection or tracking. On the other hand, label-free SERS detection and quantification demand high SERS EF and improved SERS signal reproducibility. Particularly, for label-free detection of various types of molecules and biomacromolecule species, which are of varied sizes and mostly larger, featuring low Raman cross section, the SERS substrate affinity dictates the molecules’ orientation in the 10 nm intense hot-spot zone. Critically evaluating such scenarios is out-of-the scope of this review. This review will thereby focus on labeled SERS.

## 3 SERS Nanostructure Surface as a Limiting Factor

Often one of the primary physical factors influencing SERS signal enhancement is the net concentration or number of label molecules that can anchor onto the nanostructure. With this in mind, an instant experimental plan is to increase the added label concentration or use a label with a higher affinity for anchoring onto the gold surface. Such an approach is not always feasible. For example, increasing the label concentration can destabilize the NP colloid and lead to aggregation, thereby completely altering the SERS-labeled NP structure. An understanding of the NP collisions in the colloid and the extended Derjaguin–Landau–Verwey–Overbeek (xDLVO) theory is important to gain a better perspective of NP interactions and aggregation. On the other hand, labels with functional groups like thiols are preferred for higher affinity toward gold and higher net molecule adsorption efficiency. However, it might not be the best solution when employing multiple labels (either different affinities or various labels with similar affinities) simultaneously for multiplexed SERS. Furthermore, even if the above helps in boosting the SERS signal, understanding the factor that limits label molecule attachment is vital. The number of attached molecules is governed, importantly, by the nanostructure’s total surface area. Depending on the label concentration added, i.e., the number of label molecules available for attachment, the total nanosurface area is a limiting factor for the number of molecules that can anchor onto the NP surface and contribute to SERS. It is vital to mention that only a portion of all the molecules attached onto the nanosurface will eventually provide the majority of the SERS signal. A review by Kleinman et al.,^6^ reports that ∼25% of the SERS signal was generated by less than 0.01% of the molecules on the surface with the highest EFs possible from the nanostructure. In the reported scenario on silver substrates (Ag film-overnanosphere, AgFON), approximately 95% of the molecules participated in SERS signal enhancements of 10^4^–10^6^ while 0.01% molecules at EFs 10^9^, resulting in an average SERS EF between 10^5^–10^7^. Such a scenario is evident in all SERS substrates and more critical in SERS colloids, where electric field and NP orientations dynamically vary. So, while every molecule does not contribute to the SERS EF equally, the above justifies the importance of the number of molecules on the nanosurface. Therefore, generally, a high loading of label molecules onto the surface will ensure that the highest SERS-contributing nanozone is label-rich.

To delve deeper and investigate the optimum number of molecules, we have assumed a typical Raman label footprint of 1 nm^2^ and estimated the number of molecules that can contribute to SERS for nanostructures of different sizes, shapes, and aggregation in Table 2. So, considering the maximum number of molecules that can be anchored onto the nanosurface and the label concentration added, it can be observed that at 1 nM addition, all nanostructures are only partially utilized for molecular packing. This directly impacts the SERS EF values when employing higher label concentrations than that can be utilized by the nanostructure. In contrast, when employing typical concentrations of 1 mM, most nanostructures like nanospheres, nanocubes and nanorods with a maximum dimension of 100 nm would be overpacked; i.e., their total surface area limits the molecule attachment and hence often underestimates the SERS EF for such nanostructure and label concentration combinations. Whereas, even at 1 mM concentrations, nanostars and nanoassemblies with a maximum dimension of 100 nm featuring a higher surface area than the above-mentioned single nanostructures are not fully utilized. A concentration of 1 μM although not employed for typical SERS studies, might provide a sweet spot with an optimized high packing on the nanostructure surface and maximization of added label molecule utilization. To maximize and correctly estimate SERS EF, it is evident from further analysis as shown in Table 3 that certain nanostructures are extremely underutilized in terms of both the nanostructure surface area and number of molecules available for anchoring. Gold nanospheres of 40 nm diameter might be theoretically an optimum standard for comparison of the SERS EF across newly developed nanostructures and various Raman labels. The inclusion of such a reference standard in the multifaceted SERS research would allow for better standardization of SERS experiments and SERS EF evaluations. This suggestion should be taken with a pinch of salt mainly due to two factors. First, the NP concentrations assumed in Tables 2 and 3 are considered identical for all nanostructures and should be practically recalculated for different NP concentrations employed. And second, any form of NP aggregation needs to be eliminated as an effect of label addition by monitoring *via* techniques like absorbance (UV–visible-NIR) spectroscopy. NP aggregation can significantly alter signal enhancements by creating hot-spots (hot-spot SERS, i.e., scenario **y** instead of **x** as shown in Figure 2c) and has been discussed in detail in the next section. Detailed experimental parameters could be included in publications to strengthen the use of reference standards and enable validations across research laboratories. Nevertheless, such a readily available SERS standard, which also depicts an electric field intensity for considerable SERS measurements, could allow for standardization of SERS EF reported globally.

**Table 2:** SERS Nanostructure Shape, Size, Surface Area, and Label Anchorage Comparison at Different Label Concentrations Used for Nanostructure Functionalization

				number of Raman label molecules that can anchor on the SERS nanostructure (considering 1 nm^2^ molecule footprint)^a^		
				1 mM	1 μM	1 nM
SERS gold nanostructures (size shape)		estimated total surface area of an individual nanostructure (nm^2^)	maximum number of molecules that can anchor (100% surface utilized)	∼6 × 10^6^	∼6 × 10^3^	∼6
spheres	diameter 20 nm	1256	1256	10^3^	10^3^	6
	diameter 100 nm	31415	31415	3 × 10^4^	6 × 10^3^	6
nanocubes	20 × 20 nm	1256	1256	10^3^	10^3^	6
nanorods	10 × 20 nm	785	785	<10^3^	<10^3^	6
	10 × 100 nm	3298	3298	3 × 10^3^	3 × 10^3^	6
nanostars^20^	100 nm (∼12 nm core size and spike length of about 70–100 nm)	∼5 × 10^3^	∼5 × 10^3^	∼10^3^ −10^4^	10^3^	6
	100 nm (∼30 nm core size and spike length of about 60–70 nm)	∼10^5^	∼10^5^	10^5^	6 × 10^3^	6
nanoassemblies	20 nm NPs assembled into 100 nm cluster	∼10^7^	∼10^7^	6 × 10^6^	6 × 10^3^	6

a All nanostructure concentration assumed to be 10^10^ nanostructures/mL

**Table 3:** Screening for a SERS Nanostructure Standard for Experimental Comparability Across Groups Based on the Optimum Surface Coverage and Label Molecule Utilized

SERS gold nanostructures (size shape)		estimated total surface area of an individual NS (nm^2^)^a^	1 μM equiv to ∼6 × 10^3^ per NS	total surface area utilized (0.64 packing density)^b^	net % molecule attached as compared to molecule available
spheres	diameter 20 nm	1256	10^3^	0.510	11
	diameter 40 nm	5027	6 × 10^3^	0.764	64
	diameter 100 nm	31415	6 × 10^3^	0.122	64
nanocubes	20 × 20 nm	1256	10^3^	0.510	11
nanorods	10 × 20 nm	785	<10^3^	0.734	10
	10 × 100 nm	3298	3 × 10^3^	0.582	32
nanostars	100 nm (∼12 nm core size and spike length of about 70–100 nm)	∼5 × 10^3^	10^3^	0.128	11
	100 nm (∼30 nm core size and spike length of about 60–70 nm)	∼10^5^	6 × 10^3^	0.038	64
nanoassemblies	20 nm NPs assembled into 100 nm cluster	∼10^7^	6 × 10^3^	<0.0001	64

a All nanostructure concentration assumed to be 10^10^ nanostructures/mL

b Random close packing of soft and hard spheres at an average of 0.64.^19^ Total surface area utilized = (no. of molecules × 1 nm^2^ × 0.64)/estimated total area available in nm^2^. Net % molecule attached = [(no. of molecules × 1 nm^2^ × 0.64)/no. of molecules available, i.e., 6 × 10^3^] × 100.

Using a concentration of the Raman label that will allow approximately 64% theoretical surface coverage^19^ of the particular nanostructure surface will be most beneficial. Experimentally, the adsorption efficiency of labels could be even lower than the theoretical assumptions and would be impacted by the already present surface ligands (affinity toward gold and their concentrations or molecular packing). This directly affects the analytical SERS EF. Using the correct label concentration will allow one, first to minimize nanoparticle colloid instability, which results from excessive availability of molecules displacing the stabilizing agents, and would further need less harsh methods (like centrifugation) to remove these excess label molecules. Importantly, it will allow near-true SERS EF calculations and avoid underestimation (when using 1 mM instead of 1 μM even though the same number of molecules can anchor in both scenarios).

## 4 Creating and Controlling SERS Nanostructure Hot-Spot

The hot-spot intensity of the nanozone where the label molecules are sitting is of vital importance. Even for the simplest SERS nanostructures, i.e., the nanospheres, the hot-spot intensity is not uniformly distributed over its entire surface area. Thus, utilizing the intense electric field hot-spot nanozones is crucial in pushing the limits of SERS. The shaped nanostructures like nanorods feature such nanozones at the tips or ends than the lateral sides of the nanorods. To benefit from this phenomenon, researchers have developed methods to selectively create hot spots at nanorods’ tips.^21^

**Figure 3:**
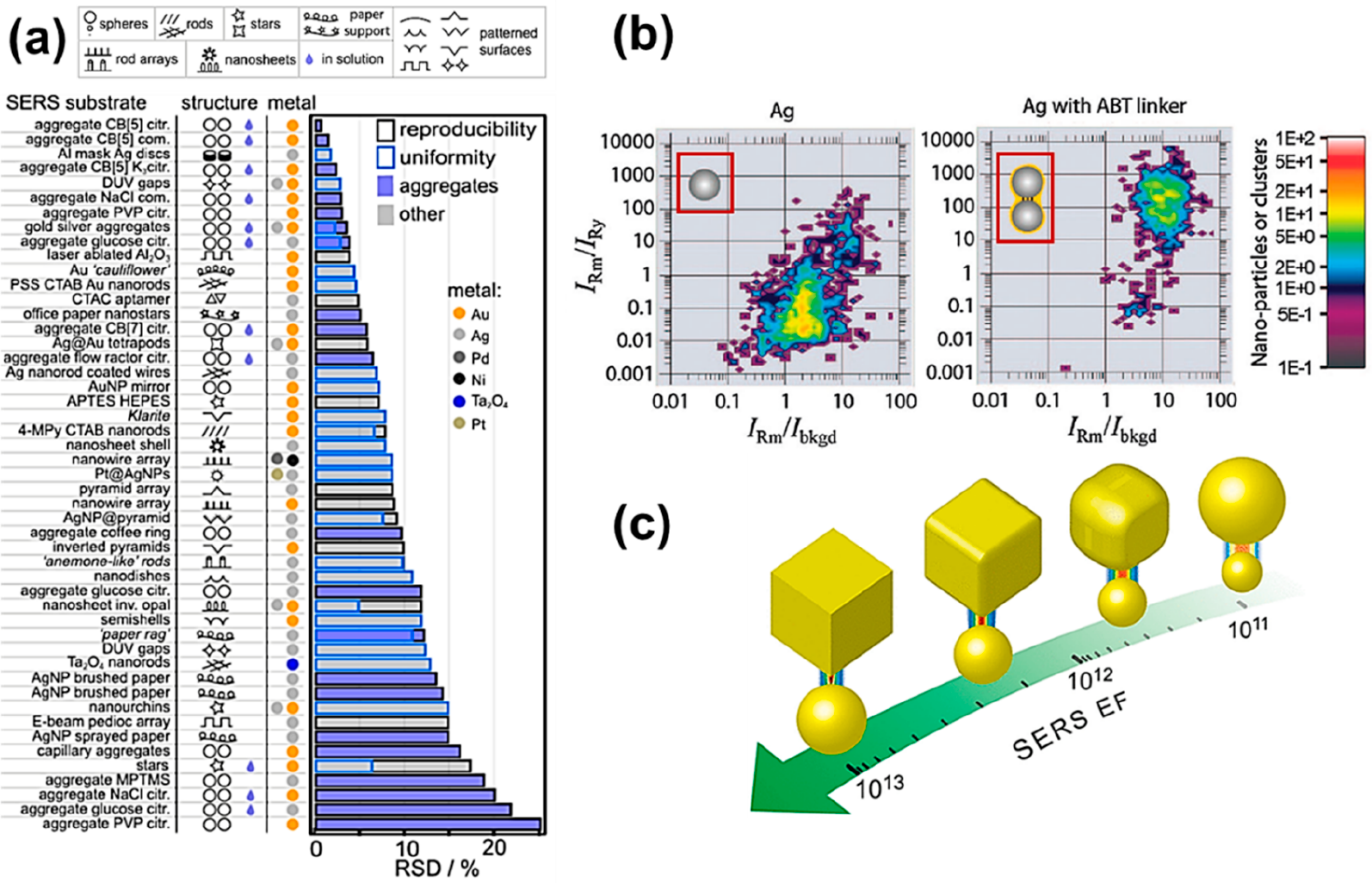
Nanoparticle aggregation and SERS EF. (a) SERS electric field at hot-spots NPs.^23^ (b) Effect of nanojunction hot-spot formation on SERS intensity (*I*_Rm_) in comparison to that of the Rayleigh intensity (I_Ry_) and background intensity (I_bkg_).^24^ (c) Tip sharpness of participating nanostructures at hot-spot impacts SERS EF.^25^ Reprinted with permission under a Creative Commons [CC-BY 4.0] from ref (23). Copyright [2021] [John Wiley & Sons Ltd.]. Reproduced from ref (24). Copyright [2016] Royal Society of Chemistry. Reproduced from ref (25). Copyright [2016] American Chemical Society.

Creating such zones with maximized electric field intensities has been a key research focus. SERS nanostructures have been developed to fulfill the above, and a summary of a few structures, along with their uniformity and reproducibility, has been shown in Figure 3a. It shows that as the nanoparticles are aggregated, creating more hot-spots,^22^ the reproducibility and uniformity of these nanostructures significantly reduce, i.e., relative standard deviation (here, RSD%) significantly increases.^23^ The simplest of the assembled nanostructures is a dimer of two nanospheres. Figure 3b shows that such a dimer features about 10^4^ times SERS intensity compared to that of the single nanostructure due to the hot-spot creation. This has been represented as the ratio of Raman Intensity to Rayleigh Intensity. The hot-spot volume depends on the curvature of the nanoparticles involved, the size of the molecule that holds or links the nanoparticles, and the interparticle distance.^24^ Thus, the above factors indirectly impact the SERS EF. The curvature of the participating nanostructures in the hot-spot significantly impacts the SERS EF, depicted in Figure 3c, which is a combined effect of the generated hot-spot volume and the inherent high electric field intensity due to the lightening rod effect of sharp-edged nanostructures.^25^

Creating the hot-spot is the tip of the iceberg. Then lies the challenge of characterizing and optimizing it and, last but not least, reproducing it with uniformity. Critical factors include the hot-spot density, its nanogap volume, and the electric field intensity at the hot-spots. The NP-NP gap and the hot-spot are governed by the surface ligand or linker molecule size for both randomly and controllably aggregated ones. Some molecular linkers that have been explored to control the interparticle distance include the star-shaped dendrimers work led by Rotello,^26^ curcubit uril led by Schermann,^23,27−30^ and multibranched polymers reported by Dey and co-workers^31−34^ which are of sizes 1–2 nm, about 1 nm, and 4–9 nm, respectively.

**Figure 4:**
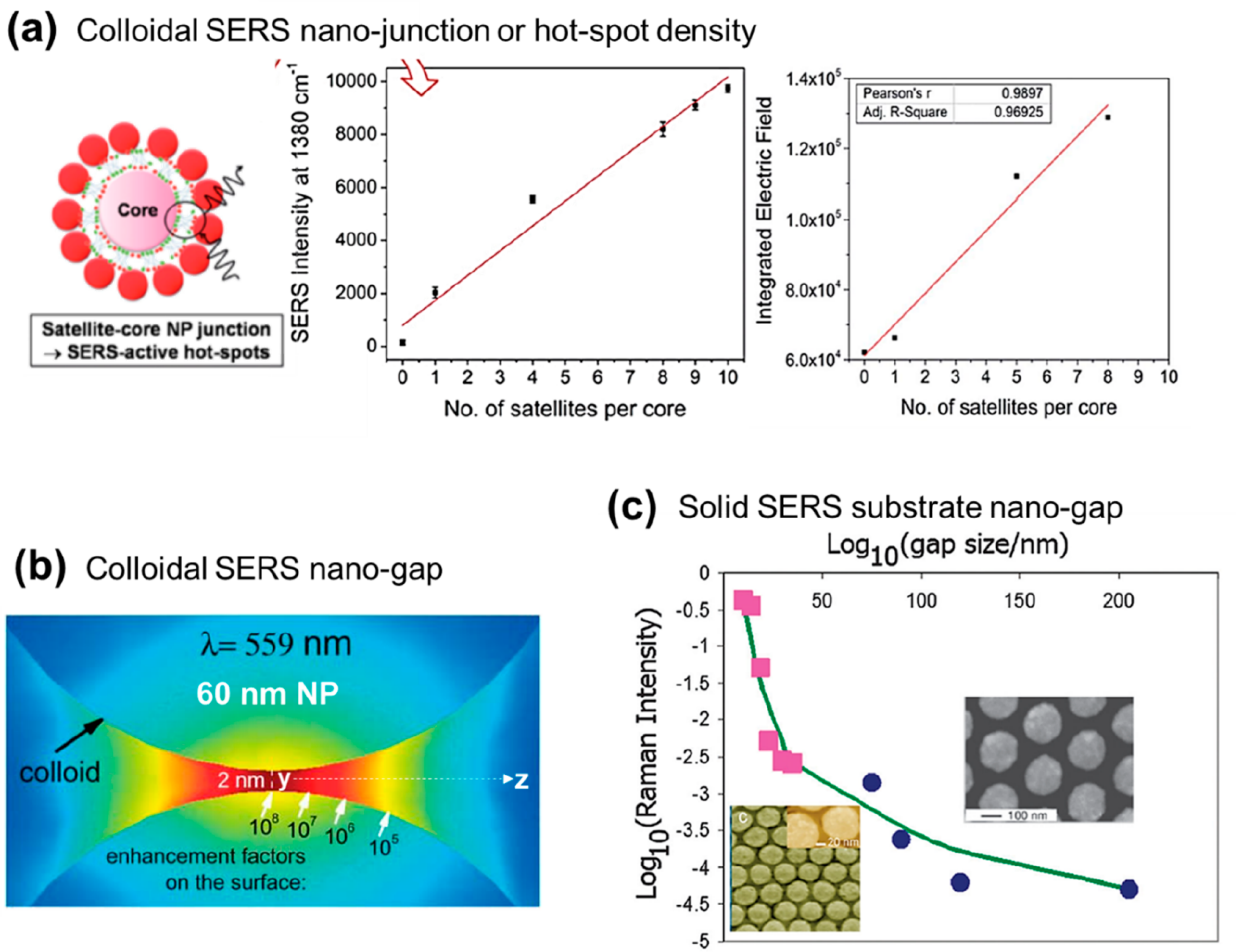
SERS nanojunction effect on SERS signal and EF. NP-NP gap. (a) SERS signal as a dependent on nanojunction or hot-spot density.^33^ (b) SERS EF as a dependent on NP-NP gap in colloid.^35^ (c) SERS signal intensity as a dependent on NP-NP gap on manufactured SERS substrates.^24^ Reproduced from ref (33). Copyright [2014] Royal Society of Chemistry. Reproduced from ref (35). Copyright [2008] Royal Society of Chemistry. Reproduced from ref (24). Copyright [2016] Royal Society of Chemistry.

Needless to say, typically, the randomly aggregated nanostructures often lack uniformity and reproducibility. Hence, developing methodologies to form optimized morphologies of nanoassemblies with control over the above parameters has been explored in a series of publications led by Dey.^31−34^ Incorporating reproducible hot-spot density into assembled nanostructures has been a challenge, which has in turn hindered SERS reproducibility. Some approaches that have shown promise have been demonstrated in core–satellite nanoassemblies where the hot-spots have been formed at the core and satellite nanojunction. Dey et al. have reported such assemblies with a reproducible number of satellites of up to 12 ± 2 per core NP, as depicted in Figure 4a. The drawback of this approach was that the satellite-to-satellite nanojunction was strategically avoided, as the satellite-to-satellite distance could not be uniformly maintained, such as not to negatively impact the hot-spot reproducibility. This limited the satellite density that could be achieved, restricting the total hot-spot density per assembly.

Although we strive to achieve reproducible SERS EF via uniform hot-spot density, the SERS EF within a hot-spot nanozone is not uniform by itself. Let us consider a simple scenario of a colloidal dimer of 60 nm NPs, as shown in Figure 4b. It is apparent that the highest SERS EF of 10^8^ is observed at the minimum interparticle distance nanozone (referred to as **y**). The same hot-spot also features a higher interparticle distance where the SERS EF drops beyond 10^3^ times if the molecule-of-interest has moved from position **y** toward **z**, as marked in Figure 4b (**y** and **z** bear similar significance as depicted in Figure 2b). For solid SERS substrates produced with even the most advanced lithographic techniques, the interparticle distance can rarely be less than 10 nm, a typical example has been shown in Figure 4c. In such cases, it is observed that the SERS intensity drops by 10^3^ times when the interparticle distance increases to 50 nm and further drops by 10^5^ at around 200 nm interparticle distances. This invariably suggests that the nanostructure curvature and linker properties could be combined to control the interparticle distance at individual hot-spots, such that they provide accessibility for labels to position themselves and become anchored at those near-uniform SERS EF hot spots.

## 5 Impact of the Physical and Functional Properties of Raman Labels on SERS EF

**Figure 5:**
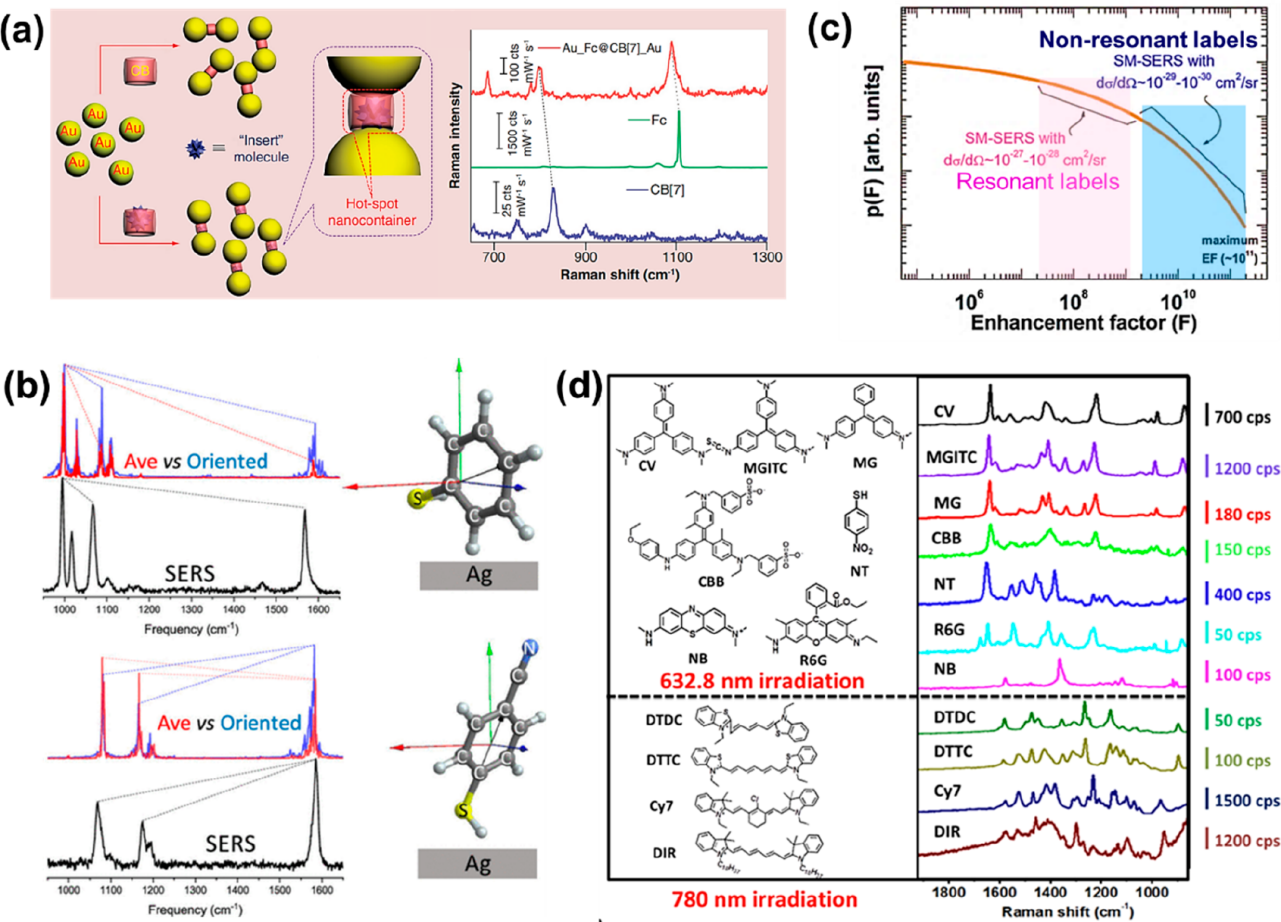
Raman labels. (a) Nanogap size control.^41^ (b) Label orientation dependent SERS intensity.^36^ (c) SERS EF for single molecule detection for resonant vs nonresonant labels.^39^ (d) SERRS intensity comparison of various resonant molecules.^40^ Reproduced from ref (36). Copyright [2019] American Chemical Society. Reproduced from ref (39). Copyright [2009] American Chemical Society. Reproduced from ref (40). Copyright [2019] American Chemical Society.

Figure 5 summarizes the key properties of Raman labels that crucially impact SERS EF. The molecular dimensions dictate if it can fit in the hot-spot nanozone (Figure 5a), and the orientation of the molecules on the plasmonic surface dictates which SERS peak positions will be enhanced^36,37^ (Figure 5b). The molecular dimension of the label also determines its footprint, which along with the functional groups dictates the orientation for packing onto the available nanosurfaces and hot-spots. For example, a Rhodamine 6G molecule is characterized as a planar molecule with dimensions of 1.1 and 1.6 nm,^38^ resulting in a footprint of 1–2 nm^2^ depending on its orientation. Whereas, a mercaptobenzoic acid 4-MBA molecule of 0.5–0.6 nm dimension would most likely be anchored to the plasmonic gold surface by the thiol end group and feature a footprint of 0.5 nm^2^. Hence, the smaller the molecular footprint, the higher density on the nanosurface, and the higher chances of finding its way to the intense hot-spot nanozones. Due to its edge-free surface, spherical nanoparticles and nanoshells have the benefit of uniform molecule packing density over its entire surface area. The nanocubes and nanorods have different crystal facets on their edges or tips than their lateral facets. This is often used to preferentially position the labels and thereby control the packing density at the tips, which typically feature higher electric field intensities than the lateral facets. In contrast, the packing density and the number of label molecules sitting at the highest electric field contributed by the sharp tips of the nanostar surface are incredibly difficult to predict and optimize experimentally.

When analyzing and reporting a SERS EF, a label with a high Raman cross-section, generally an aromatic small molecule, is utilized. Modern Raman microspectrometers can detect a signal equivalent to a cross section of 10^–20^–10^–21^ cm^2^ /sr under standard conditions. The resonance Raman cross-section of Rhodamine 6G when excited at 514 or 532 nm is of the order of 10^–24^ cm^2^/sr (see Figure 5c). For the highest SERS enhancement leading to single molecule detection, either a SMEF of only 10^3^ at its resonance is required or an SERS EF of 10^9^–10^10^ with excitation powers one million times lower than in standard conditions (i.e., in the nano-Watt range).^16,39^ Among the many available resonant Raman labels, selective ones are more efficient when excited at their resonant wavelengths. This is evident from Figure 5d where the label Malachite Green Isothiocyanate (MGITC) performs better than R6G at 632.8 nm irradiation, while at 780 nm Cyanine (Cy7) performs better than 3,3′-diethylthiadicarbocyanine iodide (DTDC).^40^ This reiterates the importance of the choice of the label utilized in both normal SERS and resonance SERS (or SERRS) experiments for reporting EFs. A molecule that exhibits low fluorescence at the laser excitation employed for this study is an additional benefit.

**Figure 6:**
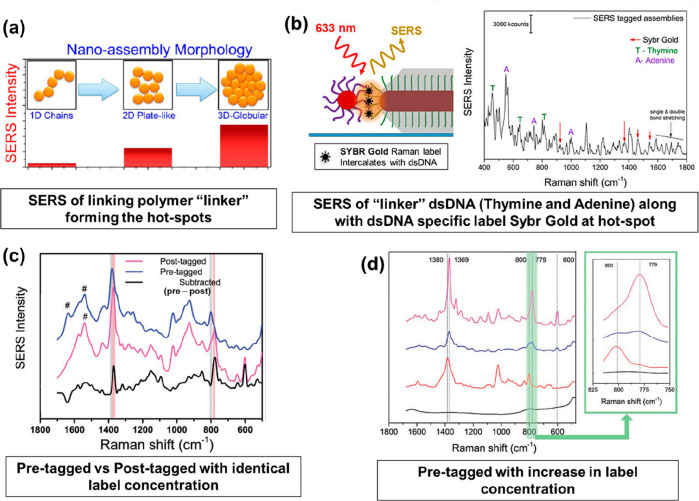
What does the hot-spot contain? (a) Hot-spots occupied by the linker molecules.^31^ (b) Both labels and linkers occupying the hot-spots.^21^ (c) Label attachment methodology (pretagging) to boost more label molecules to position themselves at created hot-spots.^44^ (d) Optimum label concentration in prehot-post forming labeling method (pretagging).^44^ Reproduced from ref (31). Copyright [2014] American Chemical Society. Reprinted with permission under a Creative Commons [CC-BY 4.0] from ref (21). Copyright [2020] [MDPI]. Adapted from ref (44). Copyright [2019] SAGE Publications.

The accessibility of labels into controlled interparticle hot-spots in nanoassemblies primarily depends on two factors. First, the accessibility of the hot-spot zone in relation to the outermost available nanosurface of the nanoassembly. Second, the linking molecule structure which can preferentially allow or hinder the labels to reach the hot-spots formed typically occupied by the linking molecules. Figure 6a shows different nanoassembly morphologies with high accessibility of labels reported for 1D nanochains and a significantly reduced accessibility to external labels for 3D globular nanoassemblies.^42^ It is noteworthy that the SERS intensity of the polymer linker (forming and occupying the hot-spots) increased with increases in hot-spot density from 1D to 3D nanoassemblies. Figure 6b depicts an example where the linking molecules are ssDNA oligomers (adenine and thymine base pairs) and sit at the most intense hot-spot of the nanorod tips. The nanoassembly formation at the tip can either be initiated by utilizing the preferential addition of linking molecules to tips rather than the nanorod lateral surface^43^ or by embedding the lateral surface in a shell with exposed tips.^21^ The linking molecule, i.e., the dsDNA was labeled with a dye to confirm that the hot-spot formed was occupied by the dye and both adenine and thymine oligomers, as observed from the SERS spectrum.^21^ The scenarios above suggests that the Raman label might not always find itself in the best spot to provide intense SERS.

To overcome the above factors, Dey and co-workers^44^ have explored a distinctly different approach for functionalizing SERS labels onto nanoassemblies, enabling a higher proportion of the labels to occupy the intense hot-spots. They refer to this as pretagging or prelabeling nanoassemblies rather than the typically used post-tagging method in literature. The terms coined to refer to when the labels or tags are incorporated, i.e., post-tagging when it is incorporated after the nanoassembly hot-spots are formed, or pretagging when the labels are introduced before the hot-spot formation. Figure 6c depicts the SERS spectra of the two scenarios with identical nanoassembly and label concentrations. The spectral difference shown as the black spectrum matches that of the SERS label employed in the study, confirming that for identical label concentration employed, a higher proportion of label molecules occupies the hot-spots using the pretagging methodology. This would boost the SERS EF further and help maximize the utilization of the formed hot-spots. In addition, the hot-spot saturation was studied in pretagged methodology, where the linking polymer concentration was kept constant to enable the formation of similar hot-spots and nanoassembly morphology. Figure 5d shows that such pretagged nanoassemblies with increasing label concentrations where the intensity of the SERS signature peaks due to the label at 779 and 1369 cm^–1^ increased in comparison to the polymer linker SERS peak at 800 and 1380 cm^–1^. It is important to note that only above a certain optimum label concentration the SERS peaks of the label become dominant over the linker SERS peaks (shown in the inset of Figure 6d). The pretagging methodology could also be extended to stepwise functionalization of single nanoparticles to improve label packing density.

## 6 Summary and Outlook

This Review critically evaluated methodologies that could contribute to maximizing SERS EF. It starts the journey by discussing the effect of the nanostructures themselves—their size and shape contributing to the surface area available for anchoring label molecules. The optimum Raman or SERS label concentration added in correlation to the nanostructure surface area involved has been discussed in great depth, as it could become a limiting factor in maximizing SERS EF. A simple SERS gold nanostructure and an optimized label concentration have also been suggested (considering the theoretical NPs/mL assumptions) that could be used as a standard reference, and its SERS EF reported under identical SERS experimental conditions. It would enable more elaborate and meaningful SERS EF value comparisons among various SERS nanostructures, labels, and between different research groups. With a critical lens, we also reviewed the SERS hot-spots– its creation, optimization, and reproducibility. The hot-spot dimensions where a high SERS signal is observed are typically when molecules sit within 0–10 nm from the nanosurface and/or when the hot-spot NP–NP gap is 0–10 nm. This demands the utilization of sub-10 nm molecules for SERS analysis. Particularly, the smaller the molecule, the better the chances of achieving high SERS and/or a sub-10 nm linker to form colloidal nanoassemblies becomes vital. The Review also iterates the impact of the molecule occupying the hot-spot in the SERS spectrum, be it the linker or the label. The key factors include the molecule’s orientation in the hot-spot, the lightening rod effect of the participating edged plasmonic nanostructures, and whether it is a resonant or nonresonant label, among others. Last but not least, the specific methods of label attachment to the nanosurface are evaluated and the impact of labeling before the hot-spot junction formation (or pretagging) as opposed to the typically employed post-tagging or post hot-spot formation. As typically reported, the label concentration directly impacts the SERS signal observable. In order to detect molecules-of-interest and improve the specificity of SERS detection, a strategy employed is guest–host complexes functionalized onto SERS nanostructures. It helps to capture specific molecules and control their positioning in the electric field, examples of guest molecules include cyclodextrins^45^ and curcubituril.^46^ Rather than being at the center of disagreements, we hope that, with the research expertise of half a century and some incredibly written reviews and perceptions, along with the methods depicted in this Review, SERS EFs can be maximized with improved reproducibility. It urgently needs to be standardized to provide the strong backbone for the SERS sensing field to become an identification and quantification sensing tool. Overcoming these would allow SERS sensors to grow in market size and eventually deliver to their full potential for solving real-world problems.
